# Tend to Compare and Tend to Be Fair: The Relationship between Social Comparison Sensitivity and Justice Sensitivity

**DOI:** 10.1371/journal.pone.0155414

**Published:** 2016-05-23

**Authors:** Shanshan Zhen, Rongjun Yu

**Affiliations:** 1 School of Psychology, Center for Studies of Psychological Application and Key Laboratory of Mental Health and Cognitive Science of Guangdong Province, South China Normal University, Guangzhou, China; 2 Department of Psychology, National University of Singapore, Singapore, Singapore; 3 Singapore Institute for Neurotechnology (SINAPSE), Center for Life Sciences, National University of Singapore, Singapore, Singapore; 4 Neurobiology/Aging programme, Center for Life Sciences, National University of Singapore, Singapore, Singapore; Middlesex University London, UNITED KINGDOM

## Abstract

Social comparison is a prerequisite for processing fairness, although the two types of cognition may be associated with different emotions. Whereas social comparison may induce envy, the perception of unfairness may elicit anger. Yet, it remains unclear whether people who tend to have a strong sense of fairness also tend to compare themselves more with others. Here, Study 1 used a modified ultimatum game (UG) and a social comparison game (SCG) to examine the relationship between justice sensitivity and social comparison sensitivity in 51 young adults. Study 2 examined self-reported social comparison and justice sensitivity in 142 young adults. Both studies showed a positive correlation between social comparison sensitivity and justice sensitivity. We reason that social comparison and justice sensitivity have an important positive correlation in human decision-making. The rejection of self-disadvantageous inequality offers may be due to the social comparison effect, which suggests that the tendency to compare oneself with others may contribute to having a strong sense of justice. Our findings suggest that the predictions of game theory may vary depending on the social culture context and incorporating notions of fairness and social comparison tendency may be essential to better predict the actual behavior of players in social interactive situations.

## Introduction

Fairness is a typical aspect of interpersonal interaction. Research on economic games suggests that people expect fairness in wealth allocation and are willing to sacrifice self-interests to punish unfair behaviors [1–4]. The ultimatum game (UG) is a classical paradigm for investigating preference for fairness [3, 5–7]. In the UG [3, 8, 9], one player, the responder, chooses whether to accept or reject a ‘take-it-or-leave-it’ reward allocation offered by another player, the proposer. That is, the proposer decides how to divide a sum of money x, for which an amount p would be kept for himself in the interval [0, x] from the total amount of money x, leaving therefore x-p for the receiver. The responder receives this information and makes a decision according to the function, f([0, x])→{‘accept’, ‘reject’}. Upon acceptance, where f(p) = ‘accept’, the resource is divided according to the offer: the proposer receives p and the responder receives x-p. Upon rejection, where f(p) = ‘reject’, both players receive nothing. According to the Nash equilibrium theory [10], the subgame perfect equilibrium predicts that proposers would normally give the responder the smallest amount possible and the responder would still accept it [11]. As such, by rejecting the offer, the responder would be worst off as s(he) will receive nothing as compared to receiving a share of the wealth even though it may be unfair. Hence, it would be better for the responder to choose to accept any demand. Given that the proposer is aware of this, he/she will likely give the smallest non-zero amount. However, in reality, responders usually reject offers lower than 20% of the total amount and the proposed offer are always between 40 and 50% of the amount [12]. As the UG is a typical dyadic bargaining situation, the proposer and the recipient must work together to earn rewards collaboratively. Cooperation could not have evolved without the sharing of payoffs. Thus, the social motives behind the rejection of unfair offers have been heatedly debated.

Some argue that rejection of unfair offers reflects inequity aversion that propels participants to equalize the payoff distribution, even if it means zero payoff [13]. Others propose a reciprocity model in which individuals could be kind or unkind to others; this model emphasizes that strong reciprocators are willing to sacrifice their own resources in order to reward fair and punish unfair behavior [14, 15]. A recent study found that the rejection of unfair offers is considered a means to avoid subjugation to the proposer [16], but this is not the case for reciprocity [2]. Indeed, fair (prosocial) and unfair (antisocial) punishers coexist in the ultimatum game [17], suggesting that there may be more than one motivation behind rejection in the UG.

One possible explanation for rejection of unfair offers is that humans often compare what they have with what others have [18]. Individuals who do not evaluate their relative status may not give much consideration to fairness. Previous research use a card game modified from a game-of-chance task to demonstrate that people have a tendency to make social comparisons [19, 20]. Many studies have provided evidence that individuals have a drive to evaluate their abilities and outcomes with regard to others [19, 21]. Recently, the UG was used to investigate the role of social comparisons between responders [4, 22, 23]. Ample findings from bargaining games showed that two bargainers make a comparison between each other in a certain context [24]. Therefore, social comparison may be the premise of any model of social preferences because many of such judgments are based on an individual’s outcomes relative to those of relevant others [13, 25, 26].

Although social comparison is an essential component of fairness sensitivity and any theory of fairness should be formulated by incorporating some kind of comparison, the precise role of trait social comparison in fairness sensitivity is not clear. For example, different emotions caused by different types of social comparison (i.e., self-advantageous comparison, self-disadvantageous comparison and equivalent comparison) can affect decision-making, such as schadenfreude and envy [27]. If some individuals tend to compare with those below them, these individuals may show stronger empathy (unhappiness at another's misfortune) or stronger schadenfreude (happiness at another’s misfortune). Similarly, individuals may feel strong envy or strong vicarious pleasure when they compare with those ahead of them. Intuitively, an individual who cares much about relative outcomes could be either a fair-minded person or a person who enjoys taking advantage of others. Thus, delineating the relationships among different types of social comparison and various types of fairness has important theoretical and pragmatic implications.

Here, we designed two tasks to investigate the relationship between social comparison sensitivity and justice sensitivity. The classic UG in which payoffs were distributed by a human proposer was adapted to measure justice sensitivity. The social comparison game (SCG, a game-of-chance task) [19, 20] was used to probe social comparison tendency. On each trial, participant’s outcome and their partner’s outcome were predetermined by a computer. Participants were asked to decide whether to keep the result of the trial (i.e., ‘save’), or to repeat the trial later (i.e., ‘again’). It meant that people had one more chance (i.e., ‘again’) to change their status when they were unsatisfied with their outcomes. In this game, social comparison was captured by measuring how risk taking (i.e., choosing ‘again’) was influenced by the relative outcomes. For individuals who did not tend to compare their own outcomes with others’ outcomes, they should only care about the absolute reward values and actively ignore what others get. Thus, if there was no social comparison effect, the choice should be influenced only by the absolute outcome participants obtained, regardless of others’ outcomes. For individuals who tended to compare themselves with others, they would actively weigh their outcomes against with others’ reward and care much about the relative values. Thus, if there were some social comparison effects, the choice should be influenced not only by absolute values but also by relative values. The SCG task provided a good measure of how much individuals actively engaged in social comparison when such comparison was readily available. The SCG measured how much individuals actively engaged the use of social comparison in making decisions. In SCG, distribution of payoffs was determined by a computer program so that rejections (i.e., ‘again’) could not be motivated by reciprocity or inequity aversion. Thus, this eliminated the possible confounds of reciprocity and inequity aversion, allowing a more systematic investigation of the social motives behind the rejection of unfair offers. If the acceptance rates of self-disadvantageous offers in UG were positively correlated with the save rates of self-advantageous comparisons in SCG, we could infer that the reason for individuals to reject unfair offers was that they tried to achieve equal outcomes between each other. If this correlation were negative, we could infer that individuals tried to get ahead of others by rejecting disadvantageous unfair offers.

The two tasks were designed to be matched in terms of reward magnitude and number of trials such that we could explore how social comparison tendency contributes to individual differences in fairness consideration. Furthermore, social comparison and justice sensitivity are considered two different personality characteristics, and different scales are used to assess social comparison [28, 29] and justice sensitivity [30, 31]. In order to further support the relationship between social comparison and justice sensitivity tendencies in the social decision making experiments, we administered the Justice Sensitivity Inventory (JSI) and the Iowa-Netherlands Comparison Orientation Scale (INCOM) to investigate whether these two personality characteristics showed the same correlations as those found in the behavioral tasks.

## Study 1

Two social games were used to determine the relationship between social comparison sensitivity and justice sensitivity as assessed by behavioral indicators. We predicted that social comparison sensitivity had a positive correlation with justice sensitivity in decision- making.

### Materials and Methods

#### Participants

Fifty-one volunteers (26 female; mean age ± SD, 20.70 ± 2.00) participated in the social decision making experiment. All participants were carefully screened for their income levels before recruitment; in order to control participants’ subjective socioeconomic status effect on their economic choices in experiments [20, 32]–only those who reported mid-range income levels were included in the study. Participants received a base payment (¥5, about $0.82) and extra earnings, depending on their performance in the task. Subjects were assigned to either the UG-SCG or SCG-UG sequence completely at random, and we found no significant effect involving experimental order (p values > 0.05). Additionally, we found no gender differences in either experiments (p values > 0.05). The study was approved by the Ethics Committee of the School of South China Normal University (SCNU). Written, informed consent was obtained from each participant, and all participants were informed of their right to discontinue participation at any time.

### Experimental paradigm

#### Ultimatum game

**Fig 1:**
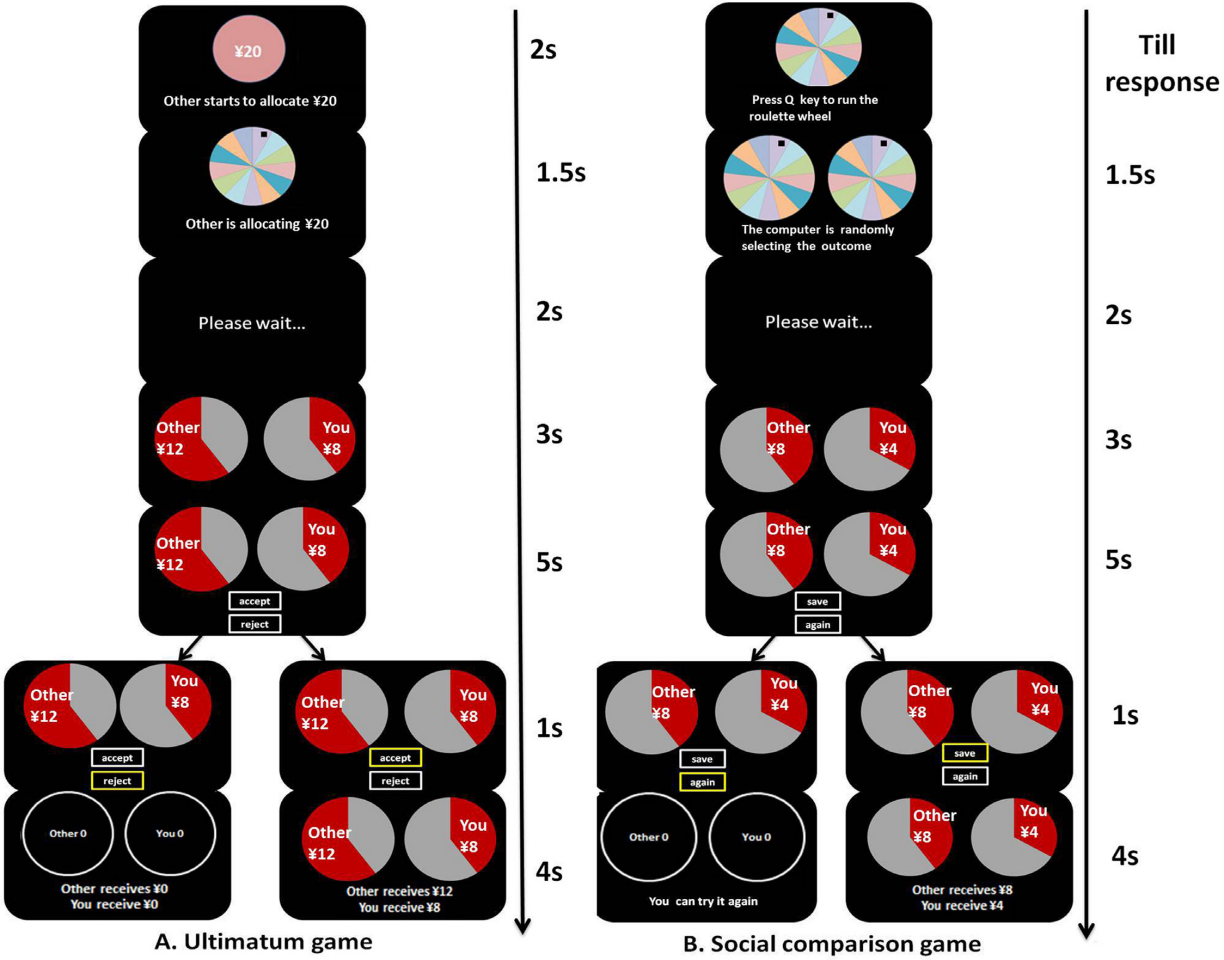
Task designs of two experiments. In the ultimatum game (A), each trial started with the presentation of a total amount of money (e.g., ¥20) which would be divided by the proposer. After 5.5 s, the monetary outcomes for both the participant and for the partner were displayed. The outcomes were presented for 3 s. Then, the participant was required to choose whether to accept the offer or reject the trial within 5 s. Finally, the feedbacks were shown according to the participant’s choice. In the social comparison game (B), each trial started with the presentation of a roulette wheel; the participant was then asked to press the Q key to make the roulette wheel run. After 3.5 s, the monetary outcomes for both the participant and for the partner were displayed. The outcomes were presented for 3 s. Then, the participant was required to choose whether to save the results or repeat the trial within 5 s. Finally, the feedbacks were shown according to the participant’s choice.

We employed the modified ultimatum game (UG) (Fig 1A) to investigate individuals’ fairness consideration. In this experiment, participants were taught the rules of UG and were assigned the role of responder. Participants were instructed that their partners had finished the division schemes between them before they participated and the division schemes were waiting for their responses. On each trial, the total amount of money allocated to the two players by a proposer, ranging from ¥4 to ¥44, was randomized and presented to the participant. Meanwhile, participants were presented with a roulette wheel for 1.5 s, which was the time window for the proposer to make the distributed decisions and for the responder to consider the response. The amount of money allocated to the proposer/responder was presented on the left/right side in the form of a pie that indicated the proportion of allocated money relative to the total amount of money. After 3 s, the participant was asked to choose either to ‘accept’ his/her partner’s division, or ‘reject’ it within 5 s. If the offer was accepted by the responder, the money was split as proposed. But if the offer was rejected, both players received nothing. Before the experiment, participants were told that one trial would be randomly chosen and implemented after the experiment. They were told that “Therefore, you should treat every trial when it appears as if it could be the one and only trial that finally determines how much you and your partner receive at the end of the experiment. Because only one trial is selected, your decisions on other trials should not in any way affect what you decide to do on the current trial.” This kind of manipulation is commonly used in research with UG [33]. Unknown to the participants, the allocation decisions were predetermined and were designed to match the outcomes in the social comparison game. Proposers’ incomes ranged from ¥0 to ¥24 in increments of ¥2. Participants’ outcomes in each trial ranged from ¥4 to ¥20 in increments of ¥4. Thus, for recipients, the exchanges could be described in terms of relative income levels (i.e., -4, -2, 0, 2, and 4) for any given absolute income level (i.e., 4, 8, 12, 16, and 20). Each combination of the two factors was presented once, resulting in a total of 25 game trials.

#### Social comparison game

We used a social comparison game (SCG) (Fig 1B) modified from a card game used in previous studies to investigate individuals’ social comparison sensitivity. Participants were instructed to play a roulette wheel game with different strangers who were not present in this task. Each trial began with a roulette wheel rather than a display of the total amount of money. Subsequently, participants were instructed to press the Q key to make their roulette wheel run with the understanding that their partner’s roulette wheel was also running. After 2 s, their income and their partner’s income were shown in two pie charts simultaneously. The red areas in the pie charts indicated the proportion of income allocated by chance relative to the maximal reward (i.e., ¥44). After 3 s, participants were asked to decide within 5 s whether to keep the result of the trial (i.e., ‘save’) or to repeat the trial later (i.e., ‘again’). They were told that the outcomes of each trial would be determined by chance. Participants were also told that the two players’ games were performed independently and participants’ outcomes or decisions would not affect their partners’ outcomes, and vice versa. If they chose ‘save,’ the assets were accepted as the computer randomly decided. If they chose ‘again,’ participants were told that they would repeat the trial later. In fact, there were no repeated trials after the game and this was explained to participants during the debriefing phase. Partners’ outcomes in each trial were presented as an amount of money ranging from ¥0 to ¥24 in increments of ¥2. Unknown to participants, their outcomes in each trial were only presented as an amount of money ranging from ¥4 to ¥20 in increments of ¥4, to achieve an equal variability of relative income levels (i.e., -4, -2, 0, 2, and 4) for any given absolute income level (i.e., 4, 8, 12, 16, and 20). Each combination of the two factors was presented once, resulting in a total of 25 game trials. The outcomes for participants and their partners were predetermined by the experimenter to control the number of trials at each absolute and relative income level, but the order of trials was pseudo-randomly determined for each participant. Before the experiment, Participants were told that one of the outcomes of all the trials would be randomly selected and added to their base payment of ¥5.

#### Post-experiment satisfaction ratings

After the end of the two experiments, participants were asked to indicate how satisfied they felt with the different types of outcomes in two experiments using an 11 point scale (from 0 most unsatisfied to 10 most satisfied).

### Results

**Fig 2:**
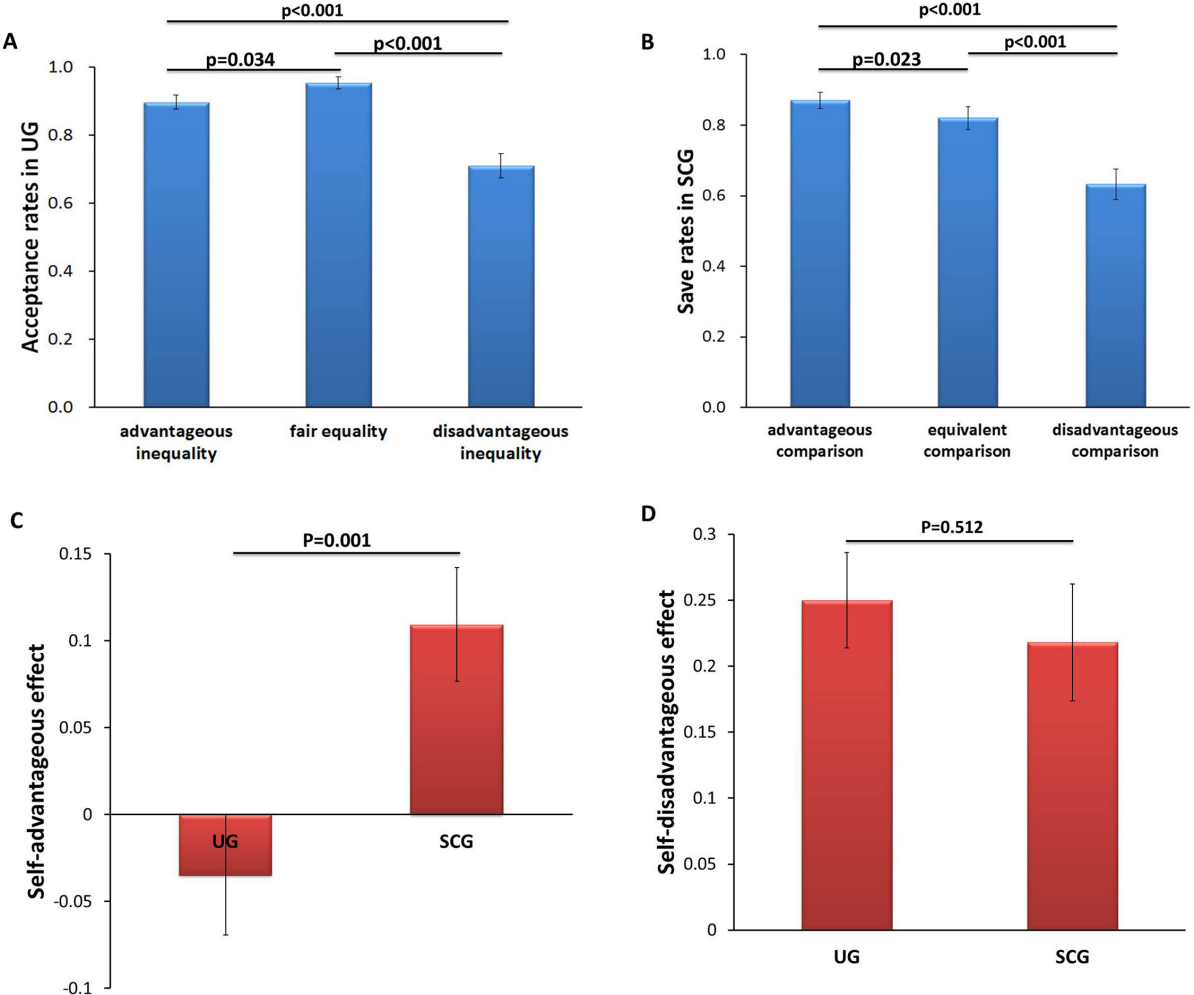
Behavioral results in Study 1. (A). Mean acceptance rates as a function of three fairness levels in the ultimatum game. (B). Mean save rates as a function of three comparison levels in the social comparison game. (C). Mean effect values as a function of self-advantageous effect (i.e., self-advantageous effect on acceptance rates in UG = (AI-FE) / FE and self-advantageous effect on save rates in SCG = (AC-EC) / EC) for ultimatum game and social comparison game. (D). Mean effect values as a function of self-disadvantageous effect (i.e., self-disadvantageous effect on acceptance rates in UG = (FE-DI) / FE and self-disadvantageous effect on save rates in SCG = (EC-DC) / EC) for ultimatum game and social comparison game. Error bars represented standard errors.

First, we tested whether the relative income levels could affect decision making in UG and SCG. In UG, the values of the relative income levels were divided into three categories: fair equality (FE; relative income level: ¥0), advantageous inequality (AI; relative income level: ¥4 and ¥2) and disadvantageous inequality (DI; relative income level: -¥4 and -¥2). Acceptance rates were calculated for each type of three fairness levels in UG. A repeated measures analysis of variance was conducted with acceptance rates of UG as the dependent variable, and fairness level as the within-subject variable. In this experiment, the Greenhouse–Geisser correction for non-sphericity was applied where appropriate. A significant main effect of fairness level (F_(2, 100)_ = 27.103, p < 0.001, η_p_^2^ = 0.352; Fig 2A) was found. Post-hoc analysis showed that the acceptance rates were significantly lower in DI condition (mean ± SE, 0.710 ± 0.036) than that in either FE condition (0.953 ± 0.017, p < 0.001) or AI condition (0.896 ± 0.020, p < 0.001), FE condition was also significantly higher than AI condition (p = 0.034). Additionally, the acceptance rates in DI condition in UG were positively correlated with AI condition in UG (Spearman correlation coefficient: r_s_ = 0.299, p = 0.033, n = 51), indicating that participants who disliked the self-disadvantageous condition also disliked the self-advantageous condition. To investigate possible learning effects during the experiment, we compared the first half with the second half of the experiment. We found no significant effects involving the session factor (first half vs. the second half), p values > 0.6.

Similar analyses were conducted for subjective satisfaction in the three conditions in UG. A significant main effect of fairness level (F_(2, 100)_ = 20.525, p < 0.001, η_p_^2^ = 0.291) was found. Participants were significantly less satisfied in DI condition (5.755 ± 0.267) than in AI (6.917 ± 0.279, p = 0.001) and FE condition (7.980 ± 0.300, p < 0.001). The self-reported satisfaction in AI condition was significantly lower than that in FE condition (p = 0.004).

In SCG, the values of the relative income levels were also divided into three categories: equivalent comparison (EC; relative income level: ¥0), advantageous comparison (AC; relative income level: ¥4 and ¥2) and disadvantageous comparison (DC; relative income level: -¥4 and -¥2). Save rates were calculated for each type of three comparison levels in SCG. Repeated measures analysis with save rates as the dependent variable revealed a significant main effect of comparison level (F_(2, 100)_ = 30.674, p < 0.001, η_p_^2^ = 0.380; Fig 2B). Further, post-hoc analysis found that the save rates were significantly higher in AC condition (0.870 ± 0.021) than that in either DC condition (0.632 ± 0.039, p < 0.001) or EC condition (0.821 ± 0.030, p = 0.023). The save rates in EC condition were significantly higher than that in DC condition (p<0.001). Additionally, the save rates in DC condition in SCG were significantly positively correlated with AC condition in SCG (r_s_ = 0.293, p = 0.037, n = 51), suggesting that participants who disliked the self-disadvantageous condition also disliked the self-advantageous condition. To investigate possible learning effects during the experiment, we compared the first half with the second half of the experiment. We found no significant effects involving the session factor (first half vs. the second half), p values > 0.5.

Similar analyses were conducted for subjective satisfaction in the three conditions in SCG. A significant main effect of comparison level (F_(2, 100)_ = 25.377, p < 0.001, η_p_^2^ = 0.337) was found. Participants were significantly less satisfied in DC condition (5.720 ± 0.279) than in AC condition (6.830 ± 0.259, p = 0.003) and EC condition (8.050 ± 0.273, p < 0.001). The self-reported satisfaction in AC condition was significantly lower than that in EC condition (p = 0.001).

In order to further characterize the effect of different relative income levels on human decision-making, we identified a self-advantageous effect and a self-disadvantageous effect to determine the choice preference in the two games. In this way, we could compare the relative acceptance rates of UG and the relative save rates of SCG in the advantageous condition or disadvantageous condition, with self-advantageous effect on acceptance rates in UG = (AI-FE) / FE and self-advantageous effect on save rates in SCG = (AC-EC) / EC; similarly, self-disadvantageous effect on acceptance rates in UG = (FE-DI) / FE and self-disadvantageous effect on save rates in SCG = (EC-DC) / EC.

**Fig 3:**
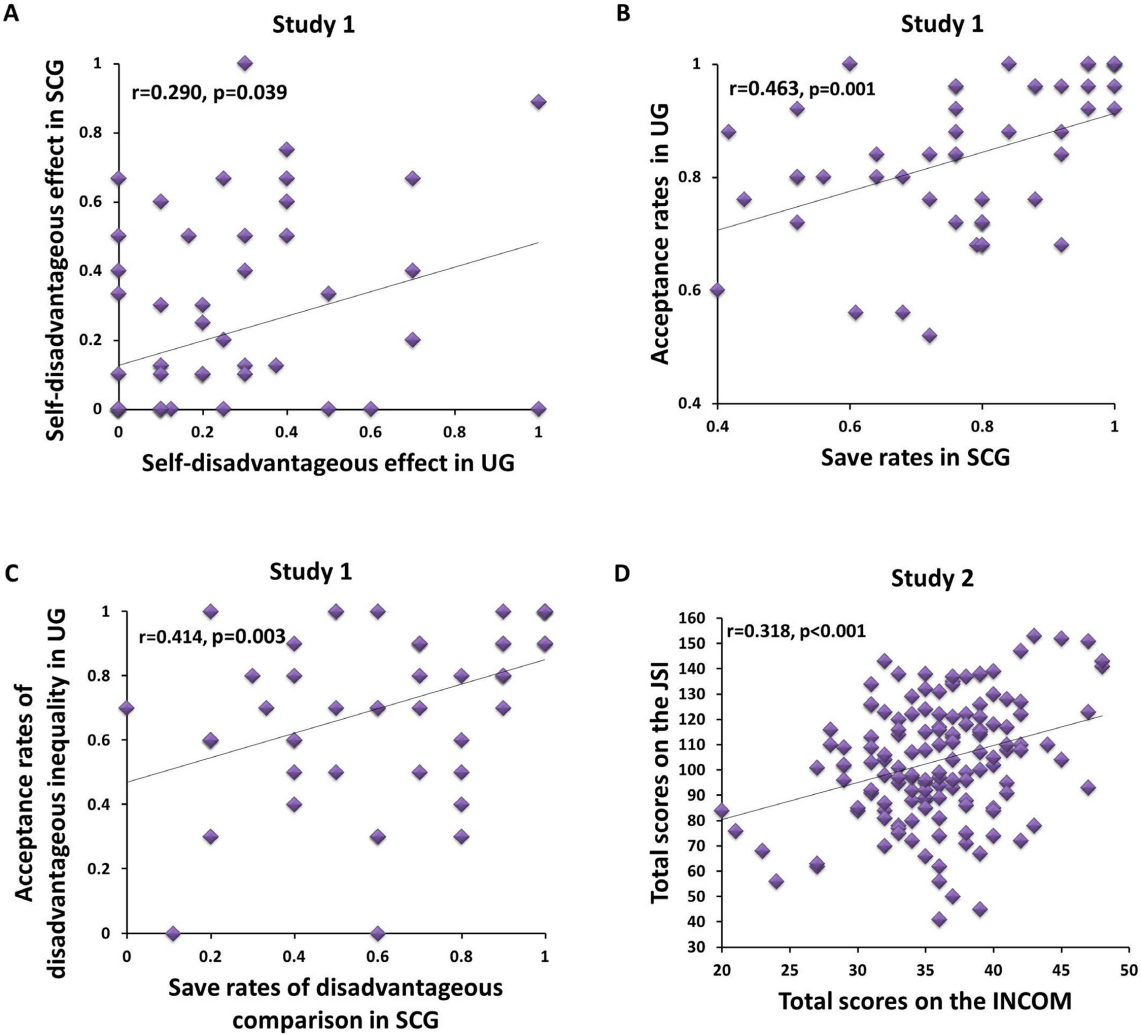
Scatter plots of the association between two experiments and between two self-reported inventories. (A). Study 1: The self-disadvantageous effect in ultimatum game had a significantly positive correlation with the self-disadvantageous effect in social comparison game. (B). Study 1: The acceptance rates in ultimatum game had a significantly positive correlation with the save rates in social comparison game. (C). Study 1: The acceptance rates in disadvantageous inequality condition in ultimatum game had a significantly positive correlation with the save rates in disadvantageous comparison condition in social comparison game. (D). Study 2: The total scores on the Iowa-Netherlands Comparison Orientation Measure had a significant positive correlation with the total scores on the Justice Sensitivity Inventory. The solid line was the regression line through the data in each plot.

A two-way repeated measures ANOVA examined the dependent measure of effect value (i.e., the value in the equation of self-advantageous effect or self-disadvantageous effect), with effect type (i.e., self-advantageous effect and self-disadvantageous effect) and experiment type (i.e., UG and SCG) as the within-subject variables. The analysis revealed a significant interaction between effect type and experiment type (F_(1, 50)_ = 8.345, p = 0.006, η_p_^2^ = 0.143). Further, post-hoc analysis showed that the effect value of SCG (0.109 ± 0.033) was significantly higher than that of UG in the self-advantageous effect type (-0.035 ± 0.035, p = 0.001; Fig 2C). However, in the self-disadvantageous effect type, the effect value of UG (0.250 ± 0.036) and SCG did not significantly differ (0.218 ± 0.044, p = 0.512; Fig 2D). A main effect of effect type was significant (F_(1, 50)_ = 18.239, p < 0.001, η_p_^2^ = 0.267). There was a marginally significant main effect of experiment type (F_(1, 50)_ = 3.117, p = 0.084, η_p_^2^ = 0.059). The self-disadvantageous effect in UG was significantly correlated with the self-disadvantageous effect in SCG (Pearson correlation coefficient: r = 0.290, p = 0.039; Fig 3A). However, no significant correlation was found between the self-disadvantageous effect in UG and the self-advantageous effect in SCG (r_s_ = 0.018, p = 0.902).

To further explore the relationship between social comparison and justice sensitivity in different conditions, we conducted correlation tests on two behavioral responses. The save rates in SCG and acceptance rates in UG were moderately positively correlated (r = 0.463, p = 0.001, n = 51; Fig 3B). Importantly, the acceptance rates in DI condition in UG were significantly positively correlated with the save rates in three conditions in SCG (i.e., DC condition, r = 0.414, p = 0.003, n = 51, Fig 3C; AC condition, r_s_ = 0.323, p = 0.021, n = 51; EC condition, r_s_ = 0.479, p = 0.001, n = 51). The acceptance rates in DI condition in UG were significantly positively correlated with the save rates in SCG as well (r = 0.395, p = 0.004, n = 51). To assess the role of three types of social comparison in fairness sensitivity, we conducted a regression analysis with the save rates in DC condition, EC condition, and AC condition in the SCG as the independent variables, and the acceptance rates in DI condition in the UG as the dependent variable. Results showed that only the save rates in DC condition in the SCG (β = 0.414, SE = 0.120, p = 0.003) significantly predicted the acceptance rates in DI condition in the UG, F_(1, 49)_ = 10.133. p = 0.003, with the adjusted R^2^ showing that 15.4% of the dependent variance was explained by this model.

### Discussion

In this study, we used two simple decision making tasks to investigate the relationship between social comparison sensitivity and justice sensitivity. Our results showed that there was a moderately positive correlation between social comparison sensitivity and justice sensitivity in decision-making. This study thus provides evidence that individuals who care more about fairness consideration also show more preference for comparing with others, or that those who prefer to compare with others may be more equality-seeking. More specifically, the significant correction between self-disadvantageous effect in UG and SCG supports the notion that dislike of being in a disadvantageous status may lead individuals to reject disadvantageous offers in UG. The results of regression analysis were consistent with previous findings that the rejection of disadvantageous offers in UG was driven by both fairness and competitive desires (e.g., dislike of being in an inferior status relative to individual’s partner) [34–37].

## Study 2

The first study used economic games to investigate sensitivity as individual differences in how people react towards different types of injustice and social comparison. However, many researchers using self-reported inventories suggest that social comparison sensitivity and justice sensitivity can also predict people’s behavior [38–40]. For instance, Justice Sensitivity inventory (JSI) has been shown to be a stable and consistent personality measure that predicts how and when people react towards engaged in or witnessed injustice [30, 41, 42]. And the Scale of Social Comparison Orientation (Iowa-Netherlands Comparison Orientation Measure, INCOM) evaluates individual differences in the tendency to compare one’s accomplishments, one’s situation, and one’s experiences with those of others [43]. Many studies have used such measure of self-other comparison orientation as a predictor of how people perform in social comparison context [28]. Thus, we used the JSI and INCOM to further analyze the relationship between the psychometric properties of individuals’ justice sensitivity and social comparison sensitivity. We predicted that both personality characteristics had a positive correlation as the results of experimental games.

### Materials and Methods

#### Participants

One hundred and fifty-one participants (52 male; mean age ± SD, 20.15 ± 1.83) were recruited to participate in Study 2. Among these participants, 45 individuals had also participated in Study 1. Participants received ¥10 for completing the questionnaires. 9 female subjects were removed from the analyses because of missing responses on some items of the scales (i.e., JSI excluded 8 subjects; INCOM excluded 1 subject), leaving a final sample of n = 142 for analysis. The study was approved by the Ethics Committee of the School of SCNU. Written, informed consent was obtained from each participant, and all participants were informed of their right to discontinue participation at any time.

#### Scales

**Justice Sensitivity Inventory (JSI):** We investigated the psychometric properties of a self-reported inventory for measuring individual differences in justice sensitivity. The Justice Sensitivity Inventory (JSI) [30] had 40 items (e.g., ‘It makes me angry when others receive a reward that I have earned.’). The Chinese version of JSI [44], standardized for mainland China participants, was adopted. The JSI includes four subscales measuring victim sensitivity (Cronbach’s α = 0.832), including ten situations to the advantage of others and to participants’ own disadvantage; observer sensitivity (Cronbach’s α = 0.892), including ten situations in which participants notice or learn that someone else is being treated unfairly, put at a disadvantage, or used; beneficiary sensitivity (Cronbach’s α = 0.852), including ten situations that turn out to participants’ advantage and to the disadvantage of others; and perpetrator sensitivity (Cronbach’s α = 0.878, n = 142), including ten situations in which participants treat someone else unfairly, discriminate against someone, or exploit someone. Each scale contained ten items that were answered on a 6-point rating scale ranging from 0 (not at all) to 5 (exactly).

**Scale for Social Comparison Orientation (INCOM, Iowa-Netherlands Comparison Orientation Measure):** We investigated the psychometric properties of a self-reported inventory for measuring individual differences in social comparison orientation. The Chinese version of INCOM [45], standardized for mainland China participants, was used. INCOM [28] had 11 items (e.g., ‘I often compare myself with others with respect to what I have accomplished in life.’). It contained two factors, the first factor, including 6 items, was labeled ‘ability.’ The second factor, labeled ‘opinions,’ comprised the remaining 5 items. Because the two types of comparison—abilities and opinions—as two sides of the same coin, the original author recommended people to use all 11 items whenever possible. Here, we only used the total scores of INCOM to investigate social comparison orientation. In the current study, the Cronbach’s alpha value for the total scale was 0.649 (n = 142). All items were answered on a 5-point rating scale ranging from 1 (disagree strongly) to 5 (agree strongly).

### Results

We investigated the relationship between JSI and INCOM across all participants. The total scores on the INCOM and the total scores on the JSI were positively correlated (r = 0.318, p < 0.001, n = 142; Fig 3D). Specifically, the scores on four JSI subscales were correlated with the total scores on the INCOM, respectively (i.e., victim: r = 0.232, p = 0.005; observer: r = 0.277, p = 0.001; beneficiary: r = 0.202, p = 0.016; perpetrator: r = 0.234, p = 0.005, n = 142).

We then examined the correlations between behavioral results and questionnaire results in participants who participated in both Study 1 and Study 2. In UG, we found that the acceptance rates in DI condition was marginally negatively correlated with the scores on victim sensitivity subscale (r = -0.280, p = 0.063, n = 45). The acceptance rates in FE condition had a positive correlation either with the scores on perpetrator sensitivity subscale (r = 0.320, p = 0.032, n = 45), or with the total scores on the JSI (r = 0.304, p = 0.042, n = 45). In SCG, no correlation was found between the save rates in three conditions and the total scores on the INCOM.

### Discussion

In this study, we used self-reported inventories to analyze the relationship between social comparison sensitivity and justice sensitivity. Our results showed that the personality characteristics of social comparison sensitivity and justice sensitivity were positively correlated. This study suggests that individuals who have a tendency to compare themselves with others may have a strong sense of justice, and vice versa. There were two key pieces of evidence that social comparison and fairness sensitivity may have a common effect in human decision-making.

First, the scores on victim sensitivity and observer sensitivity subscales were positively correlated with the total scores on the INCOM. Victim-sensitive and observer-sensitive participants are more likely to be concerned with disadvantageous inequality. Individuals who dislike disadvantageous status have a tendency to compare with others. Similarly, when individuals are concerned with advantageous inequality (i.e., beneficiary and perpetrator), they also have a tendency to compare with others. As with the fear of being exploited, observer and beneficiary sensitivity also reflect a concern for self [30]. Social comparison sensitivity may regulate the effects of perception of unfairness on outcomes and might deepen the experience of negative feeling caused by victim and observer injustice sensitivity. Even someone in advantageous status will inevitably compare the self with others, and the emotion associated with the compensation of a victim or retaliation against a perpetrator will prompt the individual to feel concern for others [30]. This possibility is consistent with the finding that the acceptance rates in DI condition in UG were significantly positively correlated with the save rates in three conditions in SCG. All the analyses reveal that social comparisons could activate the norm of equity: responders expect to be treated like others in the same circumstances [22].

Second, we found a significant correlation between victim sensitivity and the rejection rates in DI condition in UG. Victim sensitivity may promote antisocial and egoistic behavior [41]. Meanwhile, perpetrator sensitivity (i.e., benefit actively) was correlated positively with the acceptance rates in FE condition in UG. One reason why individuals high in perpetrator sensitivity behave benevolently might be that they feel guilty and have a desire to compensate the victim [30]. Our results also showed that people high in justice sensitivity accepted more equal offers in UG. These results reveal that the psychometric properties of justice sensitivity may be a relatively stable personality variable that predicts people’s fairness consideration in decision making [42, 46, 47]. Nevertheless, individual’s total scores of INCOM had no correlation with their performances in different types of comparison in SCG. One possibility is that the total scores of social comparison scale are not so sensitive to the reaction towards different types of comparison in SCG, because the scale of social comparison focuses on how much people experience social comparison (e.g., high scores in INCOM means a high comparison tendency).

In this study, the small sample size limited our ability to examine individual differences such as a gender effect. A previous study showed that women reported a level of comparison that was modest but significantly higher than that of men [28]. Other research found that women were more sensitive to justice than men [30]. Future studies could examine a possible gender difference in the relationship between social comparison and justice sensitivity with a larger sample size. Finally, to what extent our findings from laboratory experiments may apply to real life situations remains to be examined.

## General Discussion

Our main conclusion is that the rejection of self-disadvantageous inequitable offers in the UG may be due to the social comparison effect, which suggests that the tendency to compare oneself with others may contribute to having a strong sense of justice. Data from self-reported measures also supports the notion that individuals with low social comparison sensitivity may not have fairness preferences. As such, fairness may be motivated by social comparison, and social comparison may highlight the salience of fairness norms in decision-making. People are sensitive to their own outcomes relative to those of a social partner. The anticipatory negative emotion associated with gaining more or less than others may lead to a response to reject inequitable distribution. Several findings across the two experiments substantiate this conclusion.

First, the correlation between the acceptance rates in disadvantageous inequality condition in UG and the save rates in disadvantageous comparison condition in SCG suggests that when individuals recognize that they receive less than a partner, they reject unfair offers to achieve a fair distribution (zero for each). Our regression results also showed that the save rates in disadvantageous comparison condition in SCG significantly predicted the acceptance rates in disadvantageous inequality condition in UG, suggesting that fair behavior might be driven by self-disadvantageous social comparison sensitivity. One model reveals that when participants feel inferior to other players due to others’ superior status, they may experience negative emotions such as envy [19] and resentment [48] that lead them to reject the offer. Another model reveals that people reject small, unfair offers in order to avoid experiencing the emotional distress associated with accepting them [49]. Recently, one study found that impatient (short-run orientation) individuals tend to minimize their partners’ income in the UG, indicating that competitively reducing other’s payoffs is the short-run goal in ultimatum bargaining, rather than fairness [37]. Such interpretations of the rejection in disadvantageous inequality condition in UG are to some extent associated with our suggestions. That is, the rejection of self-disadvantageous inequality offers may be due to the social comparison effect. Besides, individuals reported less satisfaction with disadvantageous distribution than other distributions in both UG and SCG in our studies support these findings. In line with our findings, the self-reported data on personality traits reveals that individuals high in social comparison sensitivity care more about the victim and observer (disadvantageous inequality)—i.e., show greater injustice sensitivity—and thus reject the unequal offers in UG.

Second, we also found that individuals who rejected the disadvantageous inequality offers in UG also ‘rejected’ (did not save) the equal distributions in SCG, which supports the view that the rejection of low offers may not be a prosocial punishment. Competitive or spiteful UG punishers may try to get ahead by rejecting the offers of equivalent comparison (i.e., ‘again’) [50, 51]. There was also no correlation between the self-disadvantageous effect in UG and self-advantageous effect in SCG. Thus, it is possible that spiteful and competitive motives may also play an important role in fairness judgments [17, 50]. Punishers may have self-regarding preferences for protecting themselves against potential competitors [52].

However, the acceptance rates in disadvantageous inequality condition in UG were correlated with the save rates in advantageous comparison condition in SCG. To some extent, this finding suggests that although rejection in the disadvantageous inequality condition is a strategy for avoiding being inferior to others [16], the individual’s ultimate goal is not to be superior to others but possibly only to the extent to establish fairness between themselves and others. Given the large heterogeneity in social motives, it is possible that there are prosocial punishments in human decision making. Individuals are more likely to suppress their concern for relative advantage and make the rewards fairly, because individuals could expect partners to become envious and their relationship to be damaged [53–55]. Indeed, in our study individuals reported less satisfaction with the advantageous condition than the equivalent condition, in both UG and SCG, and this negative feeling could drive individuals to maintain social fairness norms. This possibility is in line with previous findings showing that individuals high in social comparison sensitivity might reject the unequal offer in UG due to their beneficiary and perpetrator injustice (advantageous inequality) sensitivity. However, our studies do not have adequate sample size to fully test the correlations between different types of social comparison and justice sensitivities. Future studies may identify these different subgroups and further examine the motives that would explain the association between social comparison and justice sensitivity.

It is possible that the correlation might stem from some common features such as the difference in whether subjects believed that they were really interacting with another person or not in both tasks. For example, for those participants who fully believed that they were interacting with real human partners, they might be more sensitive to our experimental manipulation (e.g., fairness and social comparison). For those who were suspicious of the experimental manipulation, they may tend to make utilitarian decisions in all tasks. If this is the case, common features such as participant's attitudes toward the tasks could also lead to significant correlations among these social tasks. Although our study cannot rule out such possibility, there is some evidence suggesting that it is unlikely that participant's general attitudes toward these tasks alone can explain our findings. A previous study used a series of experiments (i.e., the ultimatum game, the dictator game, the trust game, and the prisoner’s dilemma game) to test the correlations among the tasks. Participants were also instructed that they were unaware of their partners’ information and their decisions with each partner would not be revealed in each round [16]. If common features can contribute to correlations across social tasks, we would expect significant correlations in that study. However, researchers did not find any correlation between the participants’ tendencies to reject unfair offers in the ultimatum game and their performance in the other games, suggesting that the common features of these games would not lead to significant correlations between performances among social economic games. Future studies may explicitly measure participant's attitudes toward the tasks.

The ultimatum game is important from a socio-psychological perspective, because it illustrates the unwillingness of human beings to accept unfairness and suggests that the predictions of game theory may vary to a large extent, depending on the social-cultural context [11]. In experiments, human players care more about fairness and behave more cooperatively than strict rationality would permit. Rabin incorporated fairness into game theory and defined a concept of fairness equilibrium by adding fairness as one type of social preference [15]. Neuroimaging studies also support the notion that humans may have internal rewards for acting cooperatively in repeated interactions by showing that the reward circuits are activated when individuals engage in cooperation or punishing non-cooperative behaviors [55, 56]. Our study further highlights the importance of social comparison motive in shaping individuals’ behaviors in bargaining situations. Behavioral data have shown that respondents demand more when they know other respondents are being offered more [23, 57]. It has been found that, envy, a painful emotion activates the pain region (i.e., the anterior cingulate cortex) and schadenfreude, a rewarding reaction, activates the reward region (i.e., the ventral striatum) [27]. A recent study using a similar social comparison paradigm found that Koreans are more likely to be affected by another’s income than Americans at both the behavioral and neural levels [20]. Evidence from a meta-analysis research also showed that Asian responders have significantly higher rejection rates than responders in the US [58]. Together, these findings suggest a link between social comparison sensitivity and fairness sensitivity which is dependent on social-cultural context. Our findings provide further evidence reinforcing the notion that social comparison sensitivity plays a substantial role in determining the probability that the proposed allocation of rewards in the UG will be accepted. Incorporating notions of fairness and social comparison tendency is a promising direction and may be essential if game theory wishes to predict the actual behavior of players in social interactive situations.

It is worth noting that our social comparison task does not allow us to tease apart how much individuals seek social comparison opportunities versus how much individuals react to social comparison information. In the SCG, the social comparison information is already available and participants can choose to ignore it or weigh it heavily. There are also some situations that people can choose not to engage in social comparison situations in the first place. For example, students can choose not to inquire about others’ scores. Whether people behave differently in situations where social comparison information is available versus when it is not readily available is an interesting question. The INCOM scale tend to measure both how much people would like to seek information about others and how much people care about social comparison in general. Future studies may design novel tasks to measure social comparison seeking tendency by asking participants how much they would like to pay for information about others’ outcomes using classic willingness to pay paradigm.

In summary, different types of social comparison are positively correlated to the recipient’s fairness consideration in asset division. Specifically, the rejection of self-disadvantageous inequality offers may be due to the social comparison effect. In addition, social comparison sensitivity and justice sensitivity are two important human personality traits and these traits could predict people’s decision making in dyadic bargaining situations. However, the current methodologies have some drawbacks. First, our sample size is relatively small and self-selected student samples (volunteers) may not be representative enough. Although previous research found that self-selected student samples might have lower prosocial behavior than other populations [59], a recent study using dictator, ultimatum and trust games found that self-selected students are a representative sample for the study of social behavior [60]. Our preliminary findings need further testing with a larger and more representative sample. Second, it is not common for the proposers to propose zero reward for themselves but large rewards for the recipients. However, some studies found that recipients rejected self-advantageous offers [51, 61, 62]. Thus, it is important to investigate individual’s fair behavior in response to different types of offers in the UG. Third, there is a time limit for responding in our two tasks (i.e., 5 s), which would create a sense of time pressure and influence participant’s strategic behavior [63]. However, the average reaction time was shorter than 2 s in our two tasks, suggesting that participants had adequate time to respond. Future research may further study how time pressure impacts decision making in the UG and SCG. Fourth, in our study, participants were deceived to believe that they were playing with human partners. A common concern regarding the use of deception involves possible contamination of the participant pool. However, rejection rates in the current study replicate those typically reported from UG studies using real interacting human agencies [64]. Nevertheless, how much participants actually believed that their playing partners were real living persons may still impact how they respond in the UG. Future studies may investigate whether our results still hold if no deception was used. Another avenue of future research could focus on identifying a common neural mechanism in social comparison and fairness sensitivity. Notwithstanding these limitations, the current study is an important step forward towards understanding these important aspects of decision-making.
